# Camphor’s Therapeutic Uses and Potential Hazards: An In-Depth Review of Its Medicinal Applications

**DOI:** 10.3390/molecules31040648

**Published:** 2026-02-13

**Authors:** Anam Shabbir, Mazeyar Parvinzadeh Gashti

**Affiliations:** 1 Department of Chemistry, Forman Christian College, Lahore 54600, Pakistan; 17-26012@formanite.fccollege.edu.pk; 2 Department of Chemistry, Pittsburg State University, 1701 South Broadway Street, Pittsburg, KS 66762, USA; 3 National Institute for Materials Advancement, Pittsburg State University, Pittsburg, KS 66762, USA

**Keywords:** camphor, *Cinnamomum camphora*, biological activity, insecticidal properties, toxicity

## Abstract

Natural products have long been integral to traditional medicine, offering diverse therapeutic benefits. Increasing concerns about the side effects of synthetic drugs have heightened interest in plant-derived compounds. The camphor tree (*Cinnamomum camphora* (L.) J. Presl) and its derivatives, such as camphor oil, have been valued for centuries. Historically, *C. camphora* was used as a fumigant during the Black Death, a prized ingredient in perfumes, and a key component in embalming fluids. Today, camphor extracted from *C. camphora* is widely used as a fragrance in cosmetics, a flavoring agent in food, an ingredient in household cleaners, and a topical remedy for minor muscle pain. Camphor is primarily obtained through steam distillation of the wood but can also be synthetically produced from turpentine. Camphor exhibits a broad spectrum of biological activities, including insecticidal, antimicrobial, antiviral, anticoccidial, antinociceptive, anticancer, and antitussive effects, and has historically been employed to alleviate inflammation, congestion, pain, and irritation. This review integrates recently published research (up to 2025) on the biological activities and therapeutic applications of camphor that were not comprehensively addressed in earlier reviews. Furthermore, a mechanistic perspective is provided on camphor’s pharmacological effects, including its antibacterial, antimicrobial, antiviral, and anticancer actions, highlighting the chemical basis underlying these activities. This review provides a comprehensive overview of the history, applications, and biological properties of camphor, emphasizing its potential in preventing and treating serious diseases such as cancer and diabetes. In addition, sustainability and translational relevance are emphasized, demonstrating how camphor exemplifies the integration of traditional knowledge with contemporary medicinal research. Overall, this review offers new insights into the therapeutic potential of camphor, underscoring its promising role in addressing major medical challenges while supporting the growing importance of plant-based compounds in modern healthcare due to their effectiveness, safety, and sustainability.

## 1. Introduction

NPs have long been recognized as a cornerstone of medical advancements, offering a diverse array of bioactive compounds with significant therapeutic potential. Throughout history, various civilizations have relied on plant-based remedies to treat illnesses and maintain health. The profound impact of natural products on modern medicine is evident in the discovery of life-saving drugs such as antibiotics, anticancer agents, analgesics, and cardiovascular treatments. The vast biodiversity on Earth provides an immense reservoir of chemically unique molecules that have evolved to interact with biological systems. Many pharmaceutical agents are either directly derived from natural sources or inspired by their structural frameworks. The growing demand for plant-based therapies, particularly as alternatives to synthetic drugs with adverse effects, further emphasizes the need for scientific validation. For instance, penicillin, one of the most revolutionary antibiotics, originated from a fungal species; aspirin was derived from willow bark; and paclitaxel, a powerful anticancer drug, was extracted from the Pacific yew tree. These examples underscore the critical role of natural products in addressing diverse health challenges [1,2,3].

Natural products (NPs) continue to play a pivotal role in modern drug discovery due to their vast structural diversity, biological compatibility, and wide-ranging pharmacological activities. They have historically served as invaluable sources of lead compounds, particularly for chronic diseases such as cancer, diabetes, inflammatory disorders, and neurodegenerative conditions. Derived from plants, microorganisms, and marine organisms, NPs often exhibit high bioactivity with reduced toxicity compared to synthetic agents. Their ability to interact selectively with biological targets makes them highly at-tractive for long-term therapeutic applications, including preventive medicine, regenerative therapies, and the development of novel drug delivery systems [4]. Compared with conventional synthetic small-molecule libraries, NPs represent a more abundant and di-verse reservoir of biologically active compounds. Their complex architectures comprising multiple stereocenters, rigid ring systems, and diverse functional groups enable them to occupy broader and more intricate chemical space, often difficult to replicate synthetically. Consequently, NPs demonstrate enhanced interactions with a wide range of biological targets, leading to higher hit rates in pharmacological screening and inspiring novel scaffolds for semi-synthetic and fully synthetic drug development [5].

Although *Cinnamomum camphora* (L.) J. Presl is widely recognized as the primary natural source of camphor, this bicyclic monoterpene ketone has attracted considerable scientific interest due to its broad pharmacological potential. *Cinnamomum camphora* exhibits diverse biological activities, including antimicrobial, anti-inflammatory, analgesic, antioxidant, and neuroprotective effects, which underpin its long-standing use in traditional medicine for the treatment of respiratory ailments, pain, and infectious conditions. Camphor is primarily obtained from *Cinnamomum camphora*; in addition, it is also present in several other plant species, including *Rosmarinus officinalis*, *Artemisia* spp., *Lavandula angustifolia*, and *Ocimum basilicum*. Previous reviews have focused on the chemical properties and traditional applications of camphor as a bioactive natural compound While these studies provide valuable foundational knowledge, the present review offers novel contributions by

1. Integrating recently published research (up to 2025) on the biological activities and therapeutic applications of *Cinnamomum camphora* that were not included in prior reviews;
2. Providing a mechanistic perspective on camphor’s pharmacological effects, including anticancer, antibacterial, antimicrobial, and antiviral actions;
3. Emphasizing sustainability and translational relevance, showing how camphor exemplifies the integration of traditional knowledge with contemporary medicinal research.

By addressing these gaps, this review provides an updated, comprehensive, and critical evaluation of *Cinnamomum camphora* therapeutic potential and applications, offering insights beyond those provided in previous landmark studies [6,7,8].

Although *Cinnamomum camphora* has been traditionally used and extensively investigated in preclinical studies for various biological activities, most available evidence is derived from in vitro experiments, animal models, or ethnopharmacological observations. Robust clinical trials validating its therapeutic efficacy in humans remain limited. Therefore, the biological activities discussed in this review should be interpreted as potential or supportive evidence rather than established clinical therapies. Despite their well-established therapeutic relevance, a substantial proportion of the world’s plant biodiversity remains largely unexplored for medicinal applications. It is estimated that 250,000–400,000 plant species exist worldwide; however, only approximately 6% have been subjected to biological activity screening, and about 15% have undergone phytochemical investigation. This significant knowledge gap highlights the necessity for systematic, activity-guided research aimed at identifying novel drug leads from natural sources. Recent advances in phytochemistry, molecular biology, and bioinformatics have considerably enhanced the efficiency of natural product discovery and drug development. The increasing reliance on herbal medicines is primarily attributed to growing concerns regarding the toxicity and adverse side effects associated with conventional allopathic therapies. *Cinnamomum camphora* has emerged as an important medicinal plant owing to its broad spectrum of traditional and pharmacological applications. Notably, its essential oil exhibits pronounced chemical heterogeneity and occurs in distinct chemotypes, including camphor, linalool, borneol, 1,8-cineole, and safrole types, each characterized by a dominant constituent and specific biological activity. The indiscriminate use of the term “camphor oil” in the literature often obscures these compositional differences. In the present review, the essential oil discussed predominantly corresponds to the linalool chemotype rather than a camphor-rich oil, a distinction that is crucial for the accurate interpretation of its chemical composition and biological properties [9].

## 2. Bibliometric Analysis

To establish a robust quantitative and qualitative foundation for this review, a comprehensive bibliometric analysis of camphor-related research was conducted. Major scientific databases, including PubMed, Scopus, Web of Science, and Google Scholar, were systematically searched to retrieve peer-reviewed publications spanning the years 2012 to 2021. The search strategy employed a combination of targeted keywords and Boolean operators, including “*C. camphora*”, “camphor”, “essential oil”, “pharmacology”, “anticancer”, “antimicrobial”, and “anti-inflammatory”. Additional filters were applied to include only original research articles, reviews, and clinical studies published in English, ensuring relevance and quality of the dataset. A total of ~450 publications were retrieved and subsequently screened for relevance, excluding studies not directly related to camphor or its biological applications.

The final dataset was categorized into thematic areas, including

- Phytochemical characterization, covering essential oil composition and isolation of bioactive constituents;
- Pharmacological and bioactivity studies, focusing on mechanistic insights into anticancer, anti-inflammatory, analgesic, and cardioprotective effects;
- Antimicrobial and traditional medicine applications, highlighting efficacy against bacterial, fungal, and viral pathogens, as well as ethnopharmacological uses;
- Nanocarrier-based drug delivery systems, reflecting the emerging trend of using nanotechnology to enhance bioavailability and targeted therapeutic delivery of camphor or camphor-containing formulations;
- Clinical and translational investigations, including preclinical models and early-phase human studies.

Several highly cited review articles published in leading journals such as *Molecules*, *Phytomedicine*, and *Journal of Ethnopharmacology* further underscore the scientific relevance and expanding scope of camphor research (Table 1).

Overall, this bibliometric assessment provides a transparent, data-driven framework, delineating key research trends, evolving focus areas, and existing knowledge gaps. Such insights not only justify the need for the present comprehensive review but also offer guidance for future investigations, particularly in mechanistic pharmacology, nanocarrier-based therapeutic delivery, and clinical translation of camphor-derived bioactives. By integrating quantitative publication trends with thematic analysis, this evaluation serves as a critical foundation for understanding the trajectory and potential of camphor research over the past 9 years.

## 3. Methodology

This review was conducted through a systematic and structured literature survey to evaluate the chemical composition, chemotypic diversity, biological activities, and biomedical applications of *Cinnamomum camphora* (L.) J. Presl and its essential oils. Major databases, including Web of Science, Scopus, PubMed, Google Scholar, and ScienceDirect, were searched for peer-reviewed articles published from 2000 up to October 2025. The initial search retrieved 120 articles; after removing duplicates and screening titles and abstracts, 100 articles were assessed in full, and 92 articles were included in the final review. Search terms included combinations of keywords such as “*Cinnamomum camphora*,” “camphor oil,” “essential oil chemotypes,” “linalool chemotype,” “pharmacological activity,” “anticancer,” “antimicrobial,” “anti-inflammatory,” “nanocarriers,” and “drug delivery,” with Boolean operators applied to ensure comprehensive coverage. Only English-language articles were considered. Inclusion criteria comprised original research, systematic reviews, and authoritative reviews reporting on phytochemistry, biological activity, mechanistic insights, or biomedical relevance. Conference abstracts, non-peer-reviewed sources, and studies lacking chemical or biological characterization were excluded. Special emphasis was placed on distinguishing camphor oil chemotypes, particularly linalool-rich versus camphor-dominant variants, to ensure accurate interpretation of chemical composition and bioactivity. Data were extracted and critically analyzed with respect to plant origin, chemotype, analytical techniques, biological models, and reported mechanisms of action. The literature was categorized into phytochemical profiling, pharmacological activity, antimicrobial and traditional uses, nanotechnology-based delivery systems, and emerging biomedical applications. Furthermore, to enhance the chemical perspective of this review, the literature reporting the isolation and analysis of camphor and *Cinnamomum camphora* essential oils was also considered. Essential oils have been commonly extracted from leaves, wood, and twigs via hydro-distillation or steam distillation, often using Clevenger-type apparatus. The chemical composition, particularly camphor content, has been characterized using gas chromatography (GC), GC–mass spectrometry (GC–MS), and high-performance liquid chromatography (HPLC). Reported camphor yields vary depending on plant part, chemotype, geographic origin, and extraction method, highlighting the importance of analytical characterization for accurate chemical profiling. This approach ensures a transparent, reproducible, and comprehensive synthesis of current knowledge, supporting the scientific rationale and scope of this review.

## 4. The Importance of Systematic Phytopharmacological Evaluation of Herbal Drugs

Herbal medicines have been employed for centuries as primary therapeutic agents; nevertheless, extensive traditional use alone does not ensure their efficacy, safety, or consistency. Rigorous phytopharmacological investigation is therefore critical to scientifically substantiate herbal remedies, guarantee quality control, and support their incorporation into evidence-based medical practice. Such investigations encompass detailed phytochemical characterization, bioactivity-guided fractionation, systematic pharmacological evaluation, and thorough toxicological assessment. These approaches enable the identification of bioactive constituents, elucidation of their molecular mechanisms, and establishment of dose–response relationships, thereby providing a robust scientific foundation for the rational development and safe application of herbal therapeutics in modern medicine [10,11,12]. Differences in plant species, geographical origin, cultivation practices, harvesting times, and extraction techniques can result in considerable variability in the chemical composition and therapeutic efficacy of herbal products. In the absence of stringent standardization and thorough pharmacological validation, such formulations may demonstrate inconsistent effectiveness or provoke unintended adverse effects. Systematic scientific evaluation mitigates these issues by defining reproducible quality standards, establishing clear dose–response relationships, and determining safety profiles, thereby ensuring the reliability, efficacy, and safety of herbal medicines for clinical and therapeutic use [13,14].

Natural products have played a pivotal role in the advancement of modern medicine, serving as the foundation for numerous life-saving drugs. Many significant pharmacological discoveries have stemmed from studies on plants, leading to the development of vital medications for a wide range of diseases. Since ancient times, various civilizations have relied on herbal remedies to treat health disorders, and contemporary scientific research continues to validate the therapeutic potential of plant-derived compounds. Despite the planet’s immense biodiversity, with an estimated 250,000 to 400,000 plant species, only a small fraction has been scientifically explored for medicinal properties. Approximately 6% of these species have been thoroughly evaluated for biological activity, and only about 15% have undergone detailed phytochemical investigations. This disparity underscores a substantial research gap and highlights the urgent need for systematic, activity-guided phytopharmacological studies of herbal medicines. Given the profound influence of medicinal plants on human health, there is growing scientific interest in exploring their phytochemical and pharmacological dimensions to identify new bioactive compounds and drug candidates. Indeed, medicinal plants have contributed significantly to modern pharmacotherapy, with nearly 25% of the active ingredients in currently prescribed synthetic drugs originating from natural sources, particularly plants [4,15].

## 5. Camphor

*Cinnamomum camphora*, a bicyclic monoterpene ketone, is a naturally occurring compound derived from the wood of *Cinnamomum camphora* and related species. Although it is a well-known and historically significant source of camphor, this compound is also found in several other plants, including rosemary (*Rosmarinus officinalis*), *Artemisia* species, lavender (*Lavandula angustifolia*), basil (*Ocimum basilicum*), and others. It is widely recognized for its distinctive, penetrating aroma and diverse bioactive properties. Historically, camphor has played a significant role in medicinal formulations, religious rituals, and industrial applications. Chinese cultures have historically employed camphor as a circulatory stimulant, while the Japanese used it in torch-lighting materials. Additionally, camphor served as a fumigant during the Black Death pandemic that ravaged Europe in the 14th century, as well as during outbreaks of smallpox and cholera. It was also combined with rosewater as a perfume ingredient, sprinkled over deceased bodies prior to burial [16,17].

Its medicinal importance is attributed to its analgesic, anti-inflammatory, antimicrobial, and decongestant properties, which make it a common ingredient in topical ointments, cough suppressants, and vapor rubs. Furthermore, camphor is incorporated into cosmetics, balms, and essential oils due to its soothing effects and aromatic appeal. Beyond its pharmaceutical and cosmetic applications, camphor is extensively utilized in pest control for its insect-repellent properties. It serves as an effective deterrent against mosquitoes, moths, and other pests, rendering it valuable in both household and agricultural contexts. Additionally, camphor has applications as plasticizer in plastic production [18].

Pharmacologically, camphor exerts its effects through mechanisms such as modulation of transient receptor potential (TRP) channels, contributing to its counterirritant and analgesic properties. However, due to its potential risks, controlled usage and adherence to safety guidelines are imperative. Overall, while camphor offers significant medicinal and industrial benefits, a careful balance between its advantages and potential hazards is essential for its safe and effective application [7,8].

Camphor trees are utilized across a wide range of sectors including industrial, cosmetic, pesticidal, pharmaceutical, timber, and ornamental applications as well as for numerous cultural purposes, with their use dating back thousands of years [19]. Historically, different parts of the camphor tree including its leaves, stems, and fruits have been utilized to produce essential oils and extracts exhibiting anti-inflammatory [20] or antifungal properties [21,22]. It has also been employed for its spasmodic effects in managing circulatory and respiratory conditions. Additionally, the camphor tree serves as a valuable raw material in the cosmetics industry, being used in the formulation of perfumes, creams, and balsamic ointments [18].

Recent advances in pharmaceutical technologies have established a scientific framework for elucidating the traditional medicinal uses of camphor. Research to date indicates that its pharmacological activities are primarily attributed to its essential oils. Technological developments now allow essential oils to be extracted from multiple parts of the plant, with active components being effectively isolated and purified. Distillation remains the predominant method for essential oil extraction, enabling the detailed study and utilization of the bioactive constituents responsible for its therapeutic effects.

A wide variety of synthetic chemicals are manufactured and employed across numerous applications; however, excessive or improper use of certain synthetic agents can pose significant health risks [23]. Consequently, essential oils derived from plants present an appealing alternative to synthetic chemicals and additives, offering benefits for food safety, preservation of nutritional quality, and human health. Several constituents of camphor tree essential oils, extracted from various plant parts, exhibit strong antimicrobial activity and have been approved for use in food products. Furthermore, the growing preference in recent decades for natural plant-based products in medicine has renewed interest in essential oils as potential alternative therapeutic agents [24,25,26].

## 6. Historical Significance of Camphor

Camphor has been utilized for medicinal, cultural, and industrial purposes for several centuries, reflecting its long-standing therapeutic relevance across diverse civilizations. Historical records document the extensive use of camphor in traditional Chinese medicine (TCM), Ayurveda, Unani, and Persian medical systems, where it was valued for its cooling, antiseptic, analgesic, and anti-inflammatory properties. In TCM, camphor was commonly prescribed for the treatment of pain, inflammation, infectious diseases, and cardiac ailments, whereas Ayurvedic texts describe its application in respiratory disorders, digestive complaints, and dermatological conditions [27,28]

Beyond its therapeutic importance, camphor also held substantial economic and cultural significance, especially in East and Southeast Asia, where *C. camphora* was a principal natural reservoir of aromatic substances. For centuries, camphor was deeply embedded in local traditions, ritual practices, and early industrial applications. During the medieval era, it emerged as a highly valued commodity along major transcontinental trade networks linking Asia with the Middle East and Europe. Its high demand was driven by its intense fragrance, preservative properties, and perceived protective effects against decay and disease. Camphor was widely utilized in embalming and preservation practices, incorporated into perfumes and ointments, and used in religious and ceremonial contexts. These diverse applications elevated camphor’s commercial importance and positioned it as a strategic material in early global trade systems [29,30].

The scientific relevance of camphor increased substantially during the nineteenth century in parallel with major advances in organic chemistry. It was one of the earliest natural products to be systematically isolated, structurally characterized, and ultimately synthesized, marking a milestone in the development of stereochemistry and synthetic organic chemistry. Detailed investigations into its molecular structure and optical activity provided fundamental insights into three-dimensional molecular arrangement and chiral compounds. This shift from a traditional natural remedy to a well-defined chemical entity not only enhanced understanding of structure–activity relationships but also played a pivotal role in shaping the early principles of modern medicinal chemistry and pharmacology, thereby establishing camphor as a model compound in chemical and pharmaceutical research [31,32]. The long-standing historical utilization of camphor has therefore established a robust foundation for contemporary scientific investigations into its therapeutic potential, underlying mechanisms of action, and associated safety profiles.

### 6.1. Habitat of the Camphor Tree

**Figure 1:**
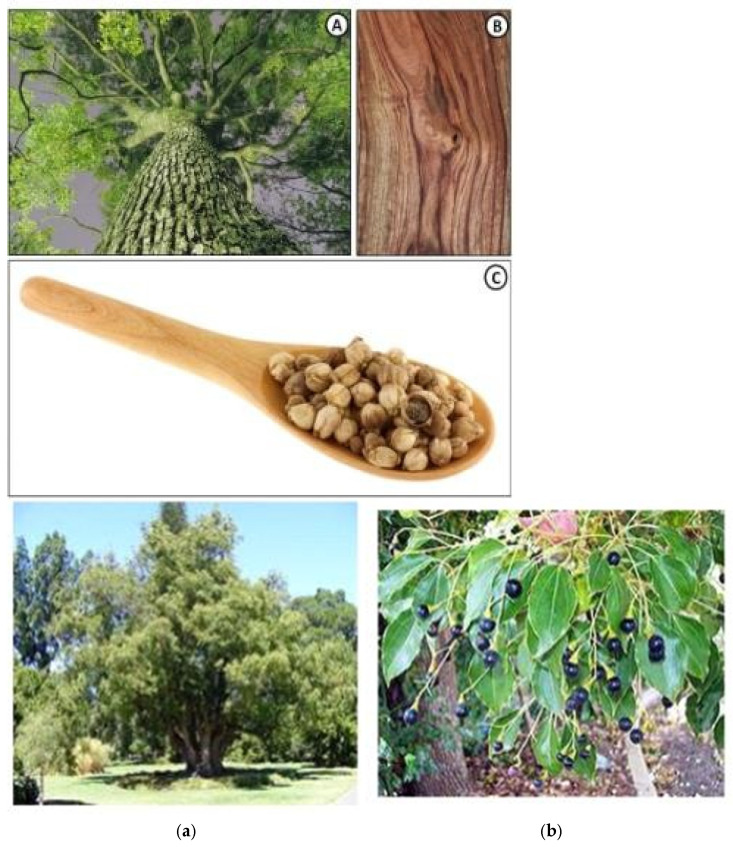
(**a**) Camphor Laurel in fruit at Turramurra railway station; (**b**) In Australia [23].

The camphor tree, *Cinnamomum camphora* (L.) J. Presl, a member of the *Lauraceae* family, is native to various parts of Asia, including Taiwan, southern Japan, eastern China, and India, and has since spread to various other regions, including Australia and the Himalayas [33,34,35]. Camphor trees can attain heights of 30–40 m and a trunk diameter of up to 3 m, typically thriving at altitudes between 900 and 2500 m above sea level. The bark is yellow to brown and exhibits vertical fissures. The leaves are alternate, displaying three or more prominent veins, and feature robust dormant buds enclosed within large, silky-textured axillary recesses [36]. This tree is among the plant species highly esteemed in Asia for its economic and cultural significance. Extensive cultivation has been established in China and Korea, where it has been officially recognized and protected as a cultural heritage resource [37]. *Cinnamomum camphora* is officially designated and protected as a natural monument in Jeju, Korea. The Korea Ministry of Culture, Sports and Tourism (KMoCST) has highlighted its long-standing use as a medicinal plant and emphasized that, beyond its significant biological properties, it holds substantial cultural value, offering insight into traditional Asian lifestyles and practices. *Cinnamomum camphora* is currently under significant threat, as rising demand and overexploitation have led to indiscriminate harvesting and a marked decline in its population, posing a potential risk of extinction if effective conservation measures are not implemented. Preserving this endangered species requires the collection and analysis of genetic diversity data, which is critical for its long-term survival. Maintaining genetic variability ensures the species’ adaptive capacity to environmental changes, and without it, the tree faces an increased risk of extinction due to diminished resilience and reduced ability to respond to ecological pressures [38]. Known for its strong, aromatic scent, the tree has been successfully cultivated beyond its native range, having been introduced and naturalized in numerous regions across the world, including parts of North America, Africa, and Australia. Typically growing to a large size, the *Cinnamomum camphora* tree features distinctive pale brown bark, which contrasts with its glossy, dark green to yellowish leaves (Figure 1). These leaves, alongside its small white flowers, are key identifiers of the tree. The flowers eventually develop into small purple berries, which serve as the plant’s fruit. Camphor trees are valued not only for their fragrant wood and leaves but also for their role in producing camphor, a bioactive compound widely used in medicinal, industrial, and cosmetic applications [39].

### 6.2. Morphological Description (Botanical Description)

Naturally found in Asian countries like Japan, Taiwan, and China, the fragrant camphor tree, scientifically known as *Cinnamomum camphora*, has also been successfully naturalized in various other regions across the world. This large tree showcases pale brown bark, dark green to yellowish leaves (refer to Figure 1), and produces small white flowers that later develop into small purple berries. Every part of the plant emits a distinct and easily recognizable camphoraceous fragrance. The essential oil, extracted from the wood (depicted in Figure 1), contains the active compound (1R)-(+)- camphor, known as natural camphor [40,41].

The leaves of *Lauraceae* are typically simple, lacking stipules, and usually arranged alternately. The flowers are radially symmetrical (actinomorphic), typically bisexual, and have a perianth composed of six sepal-like segments that are fused at the base. The androecium, the male reproductive part, often consists of four whorls of three stamens each, although the inner whorls are often sterile. The filaments of the inner whorl commonly feature enlarged glandular appendages near the base. The anthers release pollen through upwardly opening flaps, typically four in number. The pistil, the female reproductive structure, is typically single and simple, with a superior ovary containing a single ovule that hangs down in a solitary chamber. The fruit produced can be a berry or a drupe, often with a short, persistent perianth cup at the base. In contrast to other members of the Magnoliidae, the endosperm, a nutrient-rich tissue, is entirely absorbed by the embryo within the *Lauraceae* family [3]. The flowers of most species in this family are small, yellow, and aromatic. Some species have bisexual flowers containing both male and female organs. Some species have unisexual flowers, with each flower having either male organs or female organs. Some species are polygamous, in that individuals have some flowers which are bisexual, and others that are unisexual [39].

### 6.3. Physical and Chemical Properties of Camphor

Physically, camphor is a waxy, white to transparent solid that emits a characteristic, intense fragrance. It has a relatively high melting point of approximately 180 °C and exhibits sublimation at room temperature, meaning that it transitions directly from a solid to a gaseous state without passing through a liquid phase. This property contributes to its long-lasting aroma and effectiveness in medicinal and pest-repellent applications. Camphor is highly soluble in organic solvents such as alcohol, ether, and chloroform, but it is practically insoluble in water. The essential oil of *C. camphora* is chemically heterogeneous and exists in several well-defined chemotypes, including camphor, linalool, cineole, borneol, and safrole types, each characterized by a distinct major constituent and associated biological properties [42,43]. In this review, the focus is on the linalool chemotype, which is predominant in the leaves. Gas chromatography–mass spectrometry (GC–MS) analyses have identified 32 major constituents, representing approximately 97.6% of the total essential oil. The principal components include D-camphor (40.5%), linalool (22.9%), 1,8-cineole (11.3%), and 3,7,11-trimethyl-3-hydroxy-6,10-dodecadien-1-yl acetate (4.5%). Minor constituents are primarily oxygenated monoterpenes and sesquiterpenes, which contribute to the oil’s characteristic aroma and bioactivity. Accurate chemotype identification is critical, as variations in major constituents significantly influence the pharmacological and toxicological profiles of the oil. Misattribution of chemical data from unrelated species can lead to incorrect conclusions regarding biological activity; therefore, all data presented here are specific to the *C. camphora* plant. The linalool-dominant chemotype, in particular, has been associated with antimicrobial, anti-inflammatory, and antioxidant activities in preclinical studies, highlighting the importance of chemotype-specific analysis for interpreting experimental results. It should be noted that camphor occurs in multiple botanical sources, and its chemical composition and concentration can vary significantly depending on the plant species and chemotype.

**Figure 2:**
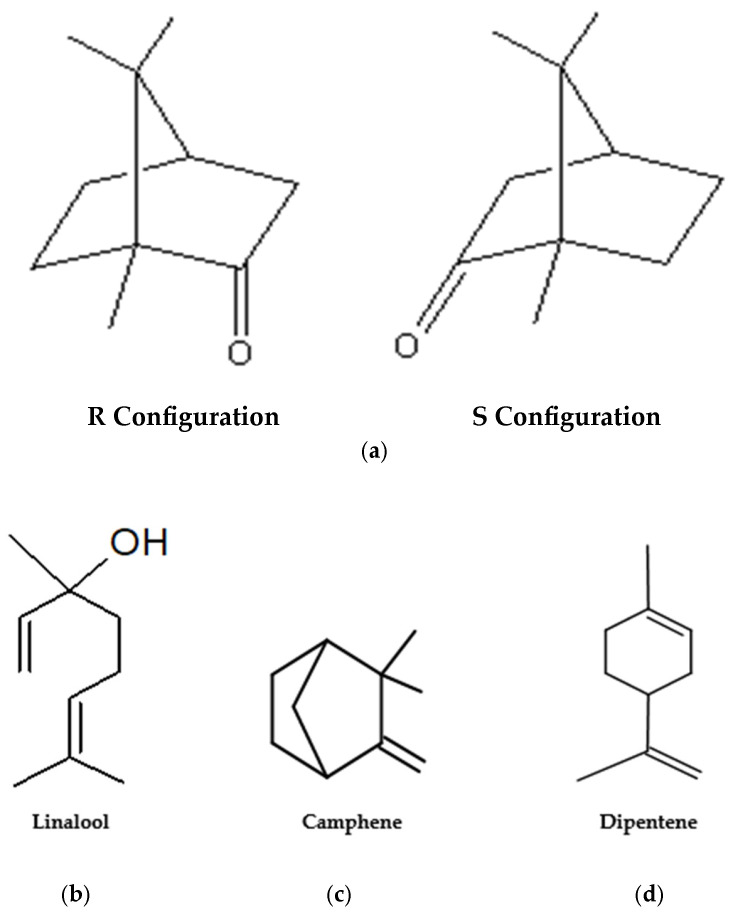

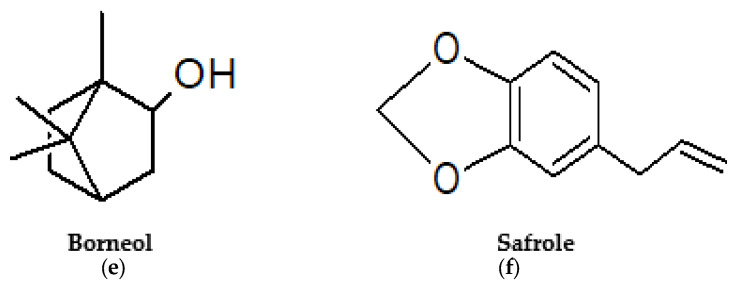
(**a**) Enantiomeric forms of camphor in *C. camphora* plant [43]. (**b**) Other chemical constituents of *C. camphora* plant [47]: linalool; (**c**) camphene; (**d**) dipentene; (**e**) borneol; (**f**) safrole.

Chemically, camphor is classified as a terpenoid ketone with the molecular formula C_10_H_16_O. Its structure consists of a bicyclic framework, specifically identified as (1,7,7-trimethylbicyclo [2.2.1]-2-heptanone). Due to its chiral nature, the *C.camphora* plant exists in two enantiomeric forms, (1S)-(−)- *C.camphora* and (1R)-(+)- *C.camphora* (shown in Figure 2), each exhibiting slightly different biological and sensory properties [44,45]. The essential oil extracted from leaves has a yield of 1.83% (*v*/*w*) and a density of 0.92 g/mL [46].

Cineole exhibits expectorant and anti-inflammatory properties, while linalool and borneol possess calming and analgesic effects. Terpineol enhances the oil’s antimicrobial activity, and pinene contributes to its respiratory benefits. The composition of camphor oil varies depending on the source and method of extraction, leading to different chemotypes such as white, brown, and yellow camphor oil. White camphor oil is the safest for medicinal use, whereas brown and yellow variants contain higher levels of safrole and other potentially toxic compounds, limiting their applications.

### 6.4. Phytochemistry

Camphor (*Cinnamomum camphora*) exists in multiple chemical varieties, each characterized by a distinct profile of essential oils [28,48]. Based on the predominant constituents of its leaf oil, the species can be classified into five chemotypes: isoborneol, camphor, 1,8-cineole, linalool, and borneol (Table 2) [49,50,51]. The phytochemical composition of different plant parts, including leaves, bark, and seeds, has been extensively studied using hydro-distillation, solvent extraction, and supercritical fluid extraction, followed by GC–MS, HPLC, FTIR, and NMR spectroscopy for both qualitative and quantitative analysis. These approaches allow the identification of monoterpenes, sesquiterpenes, and other minor constituents, while providing insights into stereochemistry, chiral purity, and essential oil variability among chemotypes. Data interpretation commonly involves comparison with authentic standards, references in the literature, and spectral databases. Collectively, these studies provide a robust chemical and phytochemical foundation for camphor’s biological activities, highlighting the importance of precise characterization for both research and practical applications.

Pragadheesh et al. [28] reported a camphor-type chemotype of *Cinnamomum camphora*, in which leaf distillates were dominated by camphor, accounting for approximately 74% of the total volatile oil composition, while the remaining major constituents collectively contributed less than 5%. In the borneol-type chemotype, Shi et al. [52] identified 11 monoterpenes, 5 sesquiterpenes, and 4 oxygenated terpenes, with (+)-borneol (66.8%) as the predominant component, followed by 1,8-cineole (4.1%), camphor (0.8%), and α-terpineol (0.4%). Oxygenated terpenes constituted the dominant fraction, representing 72.2% of the volatile oil derived from young leaves. The remaining constituents comprised monoterpenes (24.4%), primarily α-camphene, β-pinene, and β-myrcene, and sesquiterpenes (2.8%), including trans-caryophyllene, α-humulene, and γ-elemene. Chen et al. [53] reported that linalool (26.6%), 1,8-cineole (16.8%), α-terpineol (8.7%), isoborneol (8.1%), β-phellandrene (5.1%), and camphor (5.0%) were the predominant constituents of leaf essential oil. In contrast, Satyal et al. [54] demonstrated marked geographical variation in leaf oil composition. Samples collected from Makwanpur, Nepal, were rich in camphor (36.5%), followed by camphene (11.7%) and limonene (9.0%), along with moderate levels of sabinene (6.3%) and β-pinene (6.3%). Notably, leaf oil obtained from Kavre, Nepal, was composed almost entirely of camphor, accounting for approximately 98.0% of the total essential oil content.

It is important to note that both the composition and relative abundance of essential oil components vary markedly among different parts of the plant. Gyawali et al. [26] reported that the essential oil extracted from the bark contained a greater chemical diversity, with 27 identified constituents, compared to only 17 constituents detected in the fruit essential oil. The bark oil was predominantly composed of D-camphor (51.3%), followed by 1,8-cineole (4.3%), α-terpineol (3.8%), and 3-methyl-2-butenoic acid, oct-3-en-2-yl ester (3.1%). In contrast, the fruit essential oil was mainly characterized by safrole (29.0%), D-camphor (28.1%), linalool (12.8%), and 1,8-cineole (5.3%). Notably, several compounds—including γ-terpinene, isoterpinolene, 1,3,8-p-menthatriene, terpinen-4-ol, α-terpineol, eugenol, β-cadinene, and α-cubebene—were exclusively identified in the bark-derived essential oil. The biosynthesis of secondary metabolites, as well as the relative proportions of individual constituents in essential oils, is strongly influenced by environmental factors, including seasonal variation, geographical location, light intensity, surrounding vegetation and microorganisms, and soil pH. Consequently, a precise and comprehensive understanding of the phytochemical composition is critical for the effective and reliable utilization of essential oils [55].

### 6.5. Bio- and Chemical Synthesis of Camphor

Camphor can be obtained through both natural biosynthesis and chemical synthesis. Naturally, it is produced by *C. camphora* trees via the mevalonate pathway, where geranyl pyrophosphate serves as a precursor for monoterpenoid formation. Camphor is extracted from the wood through steam distillation and crystallization.

**Scheme 1:**
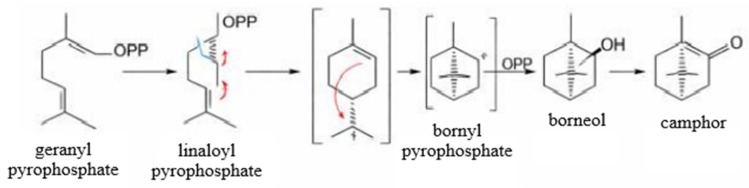
Biosynthesis of camphor [20].

Extensive research conducted by Croteau et al. [56] focused on the biosynthesis of camphor in *Salvia officinalis*. The biosynthesis of camphor initiates with geranyl diphosphate (GPP), also known as OPP, which serves as the universal 10-carbon precursor for monoterpenes [20]. GPP is produced via the mevalonate (MVA) or methylerythritol phosphate (MEP) pathways and provides both the carbon framework and the reactive diphosphate group essential for cyclization. In this pathway, (+)-bornyl diphosphate synthase catalyzes the stereospecific cyclization of GPP to form (+)-bornyl diphosphate, with the diphosphate acting as a leaving group to facilitate ring closure. Bornyl-diphosphate diphosphatase then hydrolyzes (+)-bornyl diphosphate to generate (+)-borneol, which is subsequently oxidized by (+)-borneol dehydrogenase to produce (+)-camphor (Scheme 1). The orientation and reactivity of OPP within the enzyme active site are critical for the stereochemistry and efficiency of camphor formation. Thus, GPP/OPP functions not only as the initial substrate but also as a central molecular hub, directing carbon flux toward camphor and other monoterpenes depending on enzymatic control [47].

**Scheme 2:**
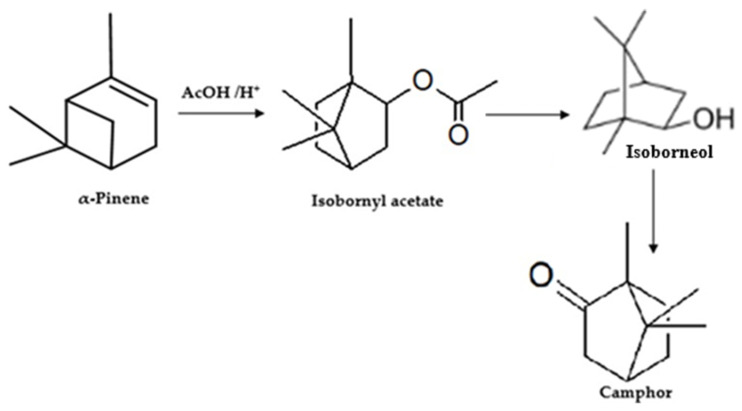
Chemical synthesis of camphor [56].

Chemically, camphor is synthesized from α-pinene, a component of turpentine oil, through oxidation and rearrangement reactions. Industrial synthesis typically involves the conversion of α-pinene to camphene, followed by oxidation to isobornyl acetate and hydrolysis to yield camphor, ensuring large-scale production for commercial applications. In the synthetic production of camphor, turpentine serves as the starting material. Through distillation, turpentine is processed to obtain α-pinene. By employing a strong acid catalyst with acetic acid as the solvent, α-pinene is transformed into camphene. Wagner–Meerwein rearrangement of camphene yields the isobornyl cation, which then reacts with acetate to form isobornyl acetate. Hydrolysis of isobornyl acetate generates isoborneol, which is further dehydrogenated to produce camphor (Scheme 2). This synthetic pathway from α-pinene results in a racemic mixture containing equal amounts of (−) and (+)-camphor [56].

Synthetic camphor is widely used in pharmaceuticals, cosmetics, and industrial applications due to its purity and controlled production, making it a cost-effective alternative to natural extraction from *C. camphora*.

## 7. Risks and Toxicity of Camphor

Although camphor offers several medicinal benefits, its use in excessive quantities can pose serious health risks. Ingesting or inhaling large amounts of camphor can result in toxicity, leading to symptoms such as nausea, vomiting, seizures, and even respiratory distress. Children are particularly vulnerable to camphor’s harmful effects, as accidental ingestion can cause severe poisoning, potentially leading to life-threatening conditions. Ingesting camphor can be fatal and has toxic effects on adults, leading to gastrointestinal tract congestion, kidney problems, and brain complications [57,58]. There is currently no specific antidote for camphor poisoning, so treatment primarily involves managing symptoms such as nausea, respiratory distress, and seizures. Once inside the body, camphor undergoes hepatic oxidative metabolism and is primarily converted into borneol and other hydroxylated camphor derivatives. This substance is then conjugated with glucuronic acid in the liver, which makes it water-soluble, allowing it to be excreted through the urine. In animal studies, such as in rats, high doses (1000 mg/kg body weight per day) have led to symptoms of toxicity, including clonic convulsions, piloerection, and decreased motility, highlighting the compound’s dangerous effects at elevated levels [59]. Camphor can irritate the skin and eyes upon direct contact, and both inhalation and skin exposure can result in acute poisoning. In a study conducted by Millet et al. [60], the toxicity of several commercially available essential oils, including sage (*Salvia officinalis*), hyssop (*Hyssopus officinalis*), thuja (*Thuja occidentalis*), and cedar (*Juniperus* and *Cupressus* spp.), was thoroughly examined. The researchers specifically focused on assessing the effects of these oils when administered to rats, aiming to determine the potential risks associated with their use. One of the most striking findings involved the administration of 3.2 g/kg of sage oil to unanesthetized rats, which resulted in severe toxic effects. The rats exhibited tonic–clonic convulsions, which are characterized by intense muscular spasms and jerking movements, followed by death. This highlighted the high level of toxicity associated with sage oil at this dosage.

In light of these dangers, regulatory bodies have implemented strict guidelines and restrictions on the concentration of camphor in consumer products. These measures aim to reduce the risk of accidental exposure and ensure that camphor is used safely in medicinal and cosmetic formulations. As a result, it is essential for users to adhere to recommended dosages and be aware of the potential side effects associated with excessive camphor exposure.

## 8. Benefits of Camphor

### 8.1. Traditional Uses of Camphor

Camphor, a naturally occurring compound obtained from *C. camphora*, has been extensively employed across pharmaceutical, industrial, and environmental applications. Historically, it has held a prominent place in Eastern cultures, where its versatile properties have been harnessed for a wide range of purposes. In traditional medicine, camphor is valued for its stimulant, antipyretic, analgesic, and anti-inflammatory effects. It has been applied to restore consciousness, reduce body heat, and alleviate pain, making it a common remedy for conditions such as fever, convulsions, stroke, respiratory distress, laryngeal discomfort, oral pain, anthrax, and ocular inflammation. Beyond its medicinal uses, camphor has also been incorporated into topical preparations, inhalants, and balms to enhance circulation, provide soothing relief, and support overall well-being, reflecting its enduring significance in both health and cultural practices [61]. *C. camphora* has a long-standing history of use in traditional Korean medicine, where it has been extensively prescribed for the management of inflammation-related conditions. Its applications are particularly noted in the treatment of rheumatism, arthritis, and other musculoskeletal disorders, owing to its anti-inflammatory and analgesic properties. Additionally, camphor preparations have been commonly applied to alleviate pain, swelling, and discomfort associated with sprains, strains, and minor injuries. Traditional formulations often include camphor in the form of oils, ointments, or balms, which are topically applied to affected areas to promote circulation, reduce inflammation, and facilitate recovery. The long-standing use of camphor in Korean medicine underscores its dual importance, encompassing both therapeutic efficacy and cultural relevance, reflecting centuries of empirical knowledge and integration into holistic approaches for maintaining musculoskeletal health and overall well-being [62].

For traditional medicinal use, approximately 30–50 g of the plant is typically consumed up to three times daily as an extract prepared with hot water and lipid-rich foods. Beyond its therapeutic applications, camphor has been utilized culturally in various regions. In Japan, small amounts of camphor were added to torches to enhance the brightness of flames, while in India, it has been commonly burned as incense in temples during religious ceremonies, valued for its aromatic smoke that is gentle on the eyes and nonirritating. These practices highlight both the practical and cultural significance of camphor across different societies, reflecting its multifunctional role in daily life and spiritual traditions [52,63].

In Ayurveda, *C. camphora* has been traditionally employed to manage a wide array of ailments, including respiratory conditions such as bronchitis and common colds, gastrointestinal disorders like diarrhea and dysentery, as well as systemic conditions such as edema, influenza, metabolic imbalances, and cardiovascular diseases. Similarly, in ancient Greek medicine, camphor was valued for its therapeutic properties as a head tonic and for supporting heart health. These historical uses reflect camphor’s broad-spectrum pharmacological potential emphasizing its enduring role in traditional medical systems across diverse cultures [64].

Camphor has long been utilized in diverse commercial and industrial applications due to its distinctive aromatic properties and chemical versatility. In the cosmetics industry, it serves as a fragrance component in perfumes, creams, and balms. It is also employed as a flavoring agent in food products and as a preservative in confectionery items to extend shelf life. Additionally, camphor functions as an insect repellent, a plasticizer in polymer formulations, and an intermediate in the synthesis of various aroma chemicals, underscoring its broad utility across multiple sectors [62,65]. Historically, camphor was employed as a fumigant during major epidemics, including smallpox and the Black Death, due to its perceived disinfectant and protective properties. During burials, bodies were often treated with rose water and camphor-infused perfumes to mask odors and reduce risk of contagion. Such practices highlight the early appreciation of camphor’s antimicrobial effects and its integral role in traditional public health measures and ritualistic preservation during periods of widespread disease [66].

### 8.2. Cardiovascular Effects (Traditional Use and Preclinical Evidence)

Camphor has been recognized for its potential cardiovascular benefits, particularly in traditional medicine. It is believed to stimulate circulation by acting as a vasodilator, helping to widen blood vessels and improve blood flow. This effect may be beneficial for individuals with poor circulation or those experiencing conditions like cold extremities or mild vascular insufficiency. Camphor’s ability to improve circulation can also promote oxygen and nutrient delivery to tissues, aiding in overall cardiovascular health.

Additionally, camphor’s mild stimulating properties can have a positive impact on heart rate. It has been used in some cultures to help regulate the heart’s rhythm, providing a calming effect in cases of arrhythmias or palpitations. However, camphor should be used cautiously, as excessive intake can lead to toxicity, potentially causing adverse cardiovascular effects such as arrhythmias, tachycardia, or low blood pressure. Therefore, while camphor offers potential cardiovascular benefits, it is important to use it within safe limits [67,68].

One of the most notable studies highlighting its therapeutic effects was conducted by Osborne, who investigated camphor’s potential to aid patients experiencing cardiac failure or collapse. These conditions are often characterized by symptoms such as cold skin, weak or absent pulses, and general circulatory collapse. In Osborne’s study, camphor was administered through subcutaneous injection, dissolved in sterile oil, and demonstrated profound improvements in patients’ conditions. The effects of camphor administration were quite striking. Following the injection, patients exhibited a marked transformation in the appearance of their skin. The once cold and pale skin became flushed, indicating an increase in blood flow to the peripheral regions of the body. This flushing was not just superficial; it was a clear indication that the camphor was effectively promoting blood circulation. The peripheral blood vessels, which had previously constricted due to circulatory failure, began to dilate. This dilation allowed blood to flow more freely, improving the efficiency of circulation and facilitating a more balanced distribution of oxygen and nutrients to vital organs and tissues.

As a vasodilator, camphor plays a key role in enhancing overall circulation. When administered during cardiac distress, it helps to relieve the symptoms of vascular insufficiency by improving blood flow to the extremities, where circulation may be reduced. This effect was particularly beneficial for patients suffering from cardiac failure, as it not only alleviated the symptoms associated with poor circulation, such as cold skin and weak pulses, but also supported the heart’s function. By dilating peripheral blood vessels, camphor helped to ease the burden on the heart, enabling it to pump more efficiently and improve the oxygenation of tissues [69].

In cases where the heart was severely weakened, camphor acted as a supportive measure to bolster the heart’s performance by alleviating some of the symptoms of heart failure. While not a cure for underlying cardiac conditions, camphor’s ability to improve circulation and blood flow made it an important adjunct in managing symptoms associated with heart failure and circulatory collapse. Its use in these contexts helped to improve the overall prognosis of patients suffering from acute or chronic cardiovascular issues, providing temporary relief and supporting the heart and other vital organs during critical times. However, it is important to note that camphor should be used with caution, as excessive doses can lead to toxicity, potentially causing serious side effects, including arrhythmias and cardiovascular complications. Nonetheless, within safe and controlled dosages, camphor has proven to be a valuable tool in improving cardiovascular function, offering vital support during instances of circulatory distress.

Singh et al. [70] study underscored the significant role of camphor in enhancing heart and peripheral circulation. Its historical use as a therapeutic agent continues to be explored and appreciated in the realm of medicine, offering valuable insights for managing conditions linked to compromised circulation and cardiovascular health [68].

Camphor has historically been used in traditional medicinal systems for its perceived stimulant and circulatory effects.

### 8.3. Potential Anticancer Activity (Preclinical Evidence)

Cancer, a complex and debilitating disease, continues to affect millions of individuals worldwide. It arises from the abnormal and uncontrolled proliferation of cells, which can occur in various tissues throughout the body. While synthetic anticancer medications have become a mainstay in treatment regimens, these drugs often carry significant adverse effects, including toxicity to healthy tissues, immune suppression, and long-term complications. This has prompted considerable interest among researchers in seeking alternative therapeutic options, particularly from natural sources. Natural compounds and their derivatives, known for their relatively lower toxicity profiles, have garnered attention as potential anticancer agents. Among the many natural compounds being investigated, camphor and menthol stand out for their promising anticancer properties. Numerous studies have focused on the in vitro examination of camphor and menthol’s effects on various cancer cell lines, aiming to assess their cytotoxic potential [71]. These studies have shown that both camphor and menthol, along with their derivatives, exhibit significant anticancer activity, selectively targeting cancer cells while sparing normal, healthy cells. Such targeted cytotoxicity is a key factor in reducing the side effects commonly associated with traditional chemotherapy drugs. The mechanisms by which camphor and menthol derivatives exert their anticancer effects involve several pathways, including induction of apoptosis (programmed cell death), inhibition of cell proliferation, and disruption of cancer cell metabolism. These compounds appear to selectively interact with cancer cells by influencing various cellular signaling pathways, which in turn triggers cell cycle arrest and promotes cell death. This makes them promising candidates for further development as anticancer agents [72].

Research into the anticancer effects of camphor and menthol derivatives is part of a larger movement towards discovering more effective, less toxic cancer treatments. By tapping into the therapeutic potential of natural compounds, scientists hope to identify novel therapies that can complement or even replace traditional cancer treatments, offering patients more tolerable alternatives with fewer side effects [73]. Camphor and menthol, with their ability to induce cancer cell death and inhibit tumor growth, represent valuable prospects for the development of new cancer therapies, and their continued investigation could lead to significant breakthroughs in cancer treatment.

Overall, the exploration of natural compounds, including camphor and menthol, as potential anticancer agents has yielded encouraging results, highlighting their cytotoxic activity against cancer cells. Further research and development in this area could potentially lead to the discovery of new, more effective and less toxic treatments for cancer patients [70].

### 8.4. Antitussive Activity of Camphor

Coughing, a prevalent clinical symptom, poses challenges for current therapies, which often provide inadequate relief. Aromatic vapors have been widely employed to alleviate symptoms of upper respiratory tract infections, primarily due to their recognized antitussive effects. In an effort to explore potential advancements in this field, researchers utilized camphor to synthesize camphor lactam (α-camphidone) through a chemical process involving hydroxylamine-O-sulfonic acid and glacial acetic acid. The resulting compounds, camphor and camphor lactam, underwent evaluation to assess their antitussive activity in guinea pigs specifically induced with cough through the administration of citric acid. The primary objective of this study was to investigate the effectiveness of camphor and its derivative, camphor lactam, in alleviating cough symptoms. Building upon the well-documented antitussive effects of aromatic vapors, researchers sought to explore the potential therapeutic benefits of these compounds, focusing specifically on their ability to counteract cough induced by citric acid [72].

The synthesis process involved the conversion of camphor into camphor lactam using hydroxylamine-O-sulfonic acid and glacial acetic acid. Subsequently, both compounds were subjected to rigorous testing using guinea pigs as the experimental model. This allowed researchers to observe and analyze the antitussive properties of camphor and camphor lactam in response to coughing induced by citric acid.

The findings of this study contribute to the understanding of the antitussive effects of aromatic compounds, particularly camphor and its derivative, camphor lactam. This research provides insights for further exploration and potential development of more effective therapies for cough management. By harnessing the potential of natural compounds, this work aims to improve symptomatic treatment options for individuals experiencing cough-related symptoms [74].

### 8.5. Antibacterial and Antifungal Activities of Camphor

The global interest in harnessing the antimicrobial properties (Table 3) of plant compounds has been steadily growing, as plants serve as a valuable source of natural products for maintaining human health. Extensive investigations have revealed the antimicrobial potential of various essential oils derived from different plant species, with camphor emerging as a major component in several of these oils. One such example is the essential oil derived from the aerial parts of sweet wormwood (*Artemisia annua*). Its composition includes significant amounts of camphor (44%), germacrene D (16%), trans-pinocarveol (11%), β-selinene (9%), β-caryophyllene (9%), and artemisia ketone (3%). Numerous studies have explored the antimicrobial activity of this essential oil and have observed noteworthy efficacy against Gram-positive bacteria, specifically Enterococcus hirae. Additionally, it has also exhibited activity against the fungi *Candida albicans* and *Saccharomyces cerevisiae*, both of which are of clinical significance. The liquid diffusion method was employed to evaluate the antimicrobial activity of the oil, revealing its ability to inhibit the growth of these microorganisms [75].

These findings highlight the potential of essential oils containing camphor, such as the one obtained from sweet wormwood, as valuable antimicrobial agents. The diverse composition of these oils contributes to their broad-spectrum activity, making them promising candidates for further exploration and development as natural alternatives to conventional antimicrobial agents. Continued research in this field may lead to the discovery of new and effective strategies for combating microbial infections. Camphor possesses well-established antimicrobial activity, corroborated by an extensive and steadily growing body of literature. Numerous studies have demonstrated its broad-spectrum efficacy against both Gram-positive and Gram-negative bacteria, as well as multiple clinically relevant fungal species. Lee et al. [76], for instance, reported pronounced antibacterial effects of camphor against major foodborne pathogens, underscoring its value as a naturally derived antimicrobial agent with potential therapeutic and preservative applications. Further investigations have elucidated several mechanistic pathways underlying its bioactivity, including disruption of microbial membrane integrity, inhibition of biofilm development, and induction of oxidative stress in pathogenic cells. Collectively, these findings strengthen the evidence base for camphor’s utility in the design of antimicrobial formulations, wound-healing biomaterials, and controlled-release delivery systems. The updated literature clearly establishes camphor as a pharmacologically significant natural compound with substantial promise for future antimicrobial and biomedical applications [77,78].

### 8.6. Insecticidal Activity of Camphor

The widespread use of synthetic pesticides has raised significant concerns due to their harmful effects on both the environment and human health. These chemicals contribute to the depletion of the Earth’s ozone layer, disrupt ecosystems, and pose long-term risks to wildlife and humans alike. In light of these dangers, there is an urgent need for more sustainable and eco-friendly alternatives. Essential oils have emerged as a promising solution due to their low toxicity to mammals, high volatility, and natural abundance, particularly in tropical regions. Furthermore, these oils are economically viable and can be produced without causing harm to the environment, making them an attractive option for replacing harmful synthetic pesticides.

One such essential oil is camphor, a compound derived from aromatic plants. Camphor has shown significant potential as an insect repellent, particularly against mosquito species such as *Anopheles culicifacies*, *Culex quinquefasciatus*, *Anopheles gambiae*, and *Anopheles funestus*. These mosquitoes are known vectors of deadly diseases like malaria and dengue, making the need for effective, non-toxic repellents critical. Studies have demonstrated that camphor effectively repels these mosquito species, offering an environmentally friendly alternative to conventional chemical repellents [79].

The essential oil extracted from *C. camphora* leaves demonstrated significant fumigant toxicity against *Lobesia serricorne* adults, with an LC_50_ value of 2.50 mg/L. This indicates that the essential oil is highly effective in inhibiting the survival of *L. serricorne* adults when exposed to it in vapor form. In a similar vein, the individual compounds isolated from the oil, namely, D-camphor and linalool, also exhibited notable fumigant toxicity against the same pest. D-camphor, with an LC_50_ value of 2.36 mg/L, was particularly potent, showing that it is a key toxicant in the oil’s fumigant action. Linalool, although slightly less effective, still demonstrated a significant LC_50_ value of 18.04 mg/L, indicating its moderate toxicity in comparison. In addition to their fumigant toxicity, D-camphor and linalool also displayed strong contact toxicity against *L. serricorne* adults [80]. The LD_50_ values for these compounds were 13.44 μg/adult for D-camphor and 12.74 μg/adult for linalool. These values suggest that both compounds are highly toxic when they come into direct contact with the insect, leading to a reduction in their survival. This highlights the potential of both D-camphor and linalool as effective natural insecticides, offering an alternative to chemical pest control methods while being derived from plant-based sources [46].

The promising repellent properties of camphor highlight its potential as a green alternative in pest control. Unlike synthetic pesticides, camphor-based products are biodegradable and have minimal environmental impact. They can be used to replace harmful chemicals that not only damage ecosystems but also pose health risks to humans and wildlife. As research and development in this field continue to grow, camphor could become a key ingredient in the formulation of safer, more sustainable insect repellents. By harnessing the power of natural compounds like camphor, we can create pest control solutions that are both effective and environmentally responsible, ensuring the protection of both public health and the planet [74].

### 8.7. Camphor as a Potential Skin Penetration Enhancer

Terpenes, which are naturally occurring compounds found in essential oils derived from plants, have been proposed as skin penetration enhancers that are considered acceptable for clinical use. Earlier research has also demonstrated that the combination of menthol and camphor enhances the ability of methyl salicylate to penetrate the skin while inhibiting its hydrolysis to salicylic acid, both in laboratory settings and in living organisms. Numerous studies have suggested that terpenes, commonly found in plant essential oils, possess properties that can facilitate the permeation of substances through the skin. This characteristic makes them attractive candidates for enhancing the delivery of therapeutic compounds. Specifically, the combination of menthol and camphor has been shown to have a synergistic effect, effectively improving the skin penetration of methyl salicylate, a widely used topical analgesic.

Notably, this combination also exhibits inhibitory properties against the enzymatic hydrolysis of methyl salicylate into salicylic acid. This is significant because the hydrolysis process can diminish the effectiveness of methyl salicylate. By inhibiting this conversion, the menthol and camphor combination helps maintain the integrity and potency of the active compound.

These findings underscore the potential of menthol and camphor as skin penetration enhancers and stabilizers in topical formulations. Further exploration and development in this field may lead to the design of more efficient transdermal delivery systems, optimizing the therapeutic benefits of various active ingredients in clinical applications. By harnessing the properties of these compounds, researchers aim to improve the effectiveness of topical treatments and enhance patient outcomes [8,40].

### 8.8. Local Anesthetic and Neuromodulatory Effects of Camphor

Camphor, when applied to a specific area of the body, can have a profound effect on the sensory nervous system, leading to a numbing or anesthetic effect. This is due to its ability to interfere with the transmission of nerve signals, reducing the sensitivity of sensory nerves and thereby alleviating pain and discomfort. As a result, camphor has shown significant promise in treating a variety of nervous disorders, particularly those that involve abnormal nerve activity such as convulsions, nervousness, epileptic seizures, and chronic anxiety. Its ability to calm and numb affected areas makes it a highly effective topical anesthetic, widely utilized in both traditional and modern medicine. The anesthetic properties of camphor stem from its interaction with specific receptors and ion channels within nerve cells, which play a crucial role in transmitting pain signals. By modulating the responsiveness of sensory nerves, camphor effectively reduces the perception of pain and discomfort in the affected area [81]. This ability to block or impair nerve conduction not only provides localized pain relief but also helps to reduce the severity of various neurological symptoms, making it a versatile compound in the treatment of disorders that affect the nervous system. Beyond its anesthetic effects, camphor also possesses muscle-relaxing properties, which can be beneficial in the treatment of muscle spasms and involuntary contractions. These symptoms are often seen in neurological conditions such as epilepsy, Parkinson’s disease, and certain types of muscular dystrophies. Camphor’s muscle-relaxant properties can help alleviate the discomfort associated with these involuntary muscle movements, improving the quality of life for individuals affected by such conditions. In addition to its pain-relieving and muscle-relaxing actions, camphor is also recognized for its calming and sedative effects on the nervous system. It has been traditionally used in herbal remedies to manage symptoms related to stress, anxiety, and nervous tension. By promoting relaxation and reducing the heightened excitability of the nervous system, camphor can help manage chronic anxiety and stress-related disorders, offering a natural alternative to pharmaceutical treatments [82].

Due to these diverse therapeutic benefits, camphor remains a valuable compound in the management of various neurological conditions [83]. Its combined effects of local anesthesia, muscle relaxation, and sedation make it a powerful tool in treating nervous system disorders, providing relief from both physical and psychological symptoms. With ongoing research into its mechanisms of action and applications, camphor holds the potential for further advancements in the treatment of nervous disorders. While camphor demonstrates efficacy as a local anesthetic, caution must be exercised in its application. Proper concentration and expert guidance are crucial to ensure safe and optimal usage. Misuse or excessive use of camphor may result in adverse effects.

In conclusion, the application of camphor locally induces a lack of sensation in sensory nerves and mitigates the severity of nervous disorders. Its role as an effective anesthetic is evident, providing relief from pain and facilitating local anesthesia. Moreover, its muscle-relaxing and calming properties contribute to its therapeutic potential in managing various neurological conditions. Further research is warranted to explore the full extent of camphor’s potential as an anesthetic agent and its application in clinical settings [62,82]. Camphor is known to produce cooling and warming sensations upon topical application, which are attributed to its interaction with sensory nerve endings and transient receptor potential (TRP) channels. These properties underlie its traditional use as a topical analgesic and counterirritant. Experimental studies indicate that camphor may exert mild local anesthetic and neuromodulatory effects; however, these actions are primarily supportive and symptomatic in nature. There is insufficient clinical evidence to support its use in the treatment of neurological disorders, and its role remains limited to topical and sensory modulation applications [84].

### 8.9. Exploratory Evidence for Camphor in Metabolic and Neurodegenerative Disorders

In recent years, camphor warrants exploration for its potential role in treating neurological disorders, particularly memory-related conditions such as Alzheimer’s disease and autism. By studying its impact on brain function and cognitive health, researchers may uncover new insights into how camphor can help alleviate symptoms or serve as a complementary therapy for such conditions. These investigations could lead to valuable advancements in medical interventions and offer new treatment options for patients with these complex disorders, ultimately improving their quality of life [76].

Recent experimental studies have explored the potential neuroprotective and metabolic effects of camphor using in vitro and animal models. These investigations suggest that camphor may influence oxidative stress pathways, inflammatory responses, and metabolic regulation. However, the available evidence is preliminary and largely restricted to laboratory-based studies. No robust clinical data currently support the use of camphor in the management of diabetes or neurodegenerative diseases such as Alzheimer’s disease. Consequently, these findings should be regarded as exploratory, highlighting areas for future research rather than confirmed therapeutic applications.

### 8.10. Camphor in Oral Care and Oral Hygiene Applications

Camphor has long been used in traditional remedies to support oral health, offering relief for a variety of issues such as dry mouth, bad breath, and throat discomfort. Its inclusion in formulations like Pan-Tambul, a popular betel leaf mixture, has made it a staple in many cultures for freshening breath and promoting oral hygiene. Camphor’s natural soothing properties help alleviate irritation and clear the throat, making it an effective option for addressing throat-related issues. By incorporating camphor into daily oral care practices, individuals can benefit from its therapeutic effects, which may help maintain optimal oral health. These benefits range from improving breath freshness to supporting overall throat health, enhancing comfort, and alleviating minor oral irritations [85].

Camphor has been traditionally incorporated into oral care formulations due to its aromatic properties and perceived antimicrobial activity. It has been used in small quantities in dental preparations to promote oral hygiene and provide a sensation of freshness. While some laboratory studies suggest antimicrobial effects against oral pathogens, clinical evidence supporting its therapeutic efficacy in oral disorders remains limited. Thus, camphor’s role in oral health is best described as supportive and traditional rather than curative.

### 8.11. Traditional and Topical Use of Camphor for Musculoskeletal Pain

Camphor oil has been a trusted remedy for centuries in the management of conditions like arthritis and rheumatism, offering a natural approach to alleviate pain and inflammation. This oil is often used topically, where its therapeutic properties can be harnessed to improve blood circulation and relieve discomfort associated with these conditions. To prepare a soothing solution, camphor oil is typically blended with carrier oils such as olive oil or sesame oil. A common mixture involves one teaspoon of camphor oil combined with 100 mL of olive or sesame oil. When applied directly to the affected areas, this blend works to stimulate blood flow, providing a sense of warmth and relaxation, which in turn can help reduce pain and stiffness in the joints. It is crucial to note that camphor oil for external use is regulated by the U.S. Food and Drug Administration (FDA), which has approved it for topical application in concentrations ranging from 3% to 11%. This regulation ensures that camphor oil, when used appropriately, is both safe and effective for pain relief. The FDA’s approval highlights its therapeutic potential and serves as a reminder to follow proper guidelines to avoid adverse reactions. Camphor-containing products are generally considered safe when used within recommended limits. According to FDA regulations, the camphor content in over-the-counter topical formulations is restricted to 3–11%. Exceeding these limits, accidental ingestion, or improper application can lead to serious adverse effects, including seizures, nausea, and central nervous system toxicity. Children are particularly vulnerable, and caregivers should strictly adhere to dosage and application guidelines. By incorporating camphor oil into a regular topical regimen, individuals dealing with arthritis or rheumatism may benefit from its anti-inflammatory and circulatory-enhancing effects. These benefits can lead to a reduction in symptoms such as joint swelling and stiffness, ultimately improving mobility and overall quality of life. Regular use of camphor oil can also help to soothe muscle aches and enhance the body’s natural healing processes, providing a gentle yet effective means of managing chronic pain and discomfort without the need for more invasive treatments [86,87,88].

Camphor has long been used topically for the relief of musculoskeletal pain associated with arthritis and rheumatism. Its counterirritant and analgesic properties are believed to provide temporary symptomatic relief by stimulating local blood flow and sensory nerves. Clinical use is generally limited to external application in balms and liniments, and its effects are primarily palliative. Current evidence supports camphor’s role in symptom management rather than disease modification or long-term therapeutic intervention.

**Table 3:** Biological activities of camphor, models or systems studied, key effects observed, and proposed mechanisms.

Biological Activity	Model or System Studied	Key Effects Observed	Proposed Mechanism	References
Cardiovascular effects	Human clinical observations; historical clinical use	Improved peripheral circulation, skin flushing, vasodilation, support in cardiac failure and circulatory collapse	Peripheral vasodilation, enhanced blood flow, and reduced cardiac workload	[66,67,68]
Anticancer activity	In vitro cancer cell lines	Selective cytotoxicity against cancer cells, induction of apoptosis, and inhibition of proliferation	Apoptosis induction, cell cycle arrest, and disruption of cancer cell metabolism	[70]
Antitussive activity	Guinea pig cough model (citric acid-induced)	Significant reduction in cough frequency	Modulation of cough reflex pathways via aromatic compounds	[22,34]
Antibacterial activity	Gram-positive and Gram-negative bacteria; foodborne pathogens	Broad-spectrum antibacterial efficacy	Membrane disruption, inhibition of biofilm formation, and oxidative stress induction	[8,53,76,77,78]
Antifungal activity	*Candida albicans*, *Saccharomyces cerevisiae*	Inhibition of fungal growth	Alteration of membrane integrity and metabolic disruption	[28,30,31]
Insecticidal activity	Mosquito species (*Anopheles*, *Culex* spp.); *Lobesia serricorne*	Strong repellent, fumigant, and contact toxicity	Neurotoxicity, respiratory inhibition, and volatility-based fumigant action	[26,50,72,74,82]
Skin penetration enhancement	In vitro and in vivo transdermal studies	Enhanced permeation of methyl salicylate; inhibition of hydrolysis	Disruption of stratum corneum lipid structure; synergistic terpene effects	[5,22]
Anesthetic and neuro- calming effects	Topical application; traditional and experimental use	Local anesthesia, pain relief, muscle relaxation, and sedative effects	Modulation of sensory nerve conduction and ion channels	[34,83,84]
Neuroprotective potential (diabetes and Alzheimer’s)	Conceptual and exploratory studies	Possible improvement in cognitive and neurological functions	Modulation of neuronal signaling and oxidative pathways	[30,75]

## 9. Drawbacks, Limitations, and Critical Properties

Camphor, despite its wide range of therapeutic applications in topical analgesics, decongestants, and antimicrobial preparations, presents several critical limitations and safety concerns that restrict its clinical usage. One major drawback lies in its narrow therapeutic window, meaning that the difference between a therapeutic and toxic dose is very small. Excessive skin absorption or accidental ingestion may lead to neurotoxicity, manifesting as seizures, confusion, respiratory depression, and even coma. In infants and young children, camphorated products pose a significant poisoning risk, prompting regulatory agencies such as the FDA to limit allowable concentrations in over-the-counter formulations [89]. Furthermore, despite its popular use as a cough suppressant and nasal decongestant, clinical evidence for camphor’s efficacy remains limited or inconclusive, leading to ongoing debates regarding its actual medicinal value.

Additional limitations are related to camphor’s volatile and lipophilic properties, which facilitate rapid absorption through the skin and mucous membranes; this enhances its pharmacological effects but simultaneously increases toxicity potential [90]. Long-term or high-dose exposure has been associated with hepatotoxicity and nephrotoxicity, raising concerns about cumulative organ damage, particularly in patients with pre-existing liver or kidney disorders [91]. Camphor also exhibits high flammability and volatility, presenting storage and handling hazards in pharmaceutical settings, while its strong odor may cause irritation and allergic reactions in sensitive individuals [92]. Collectively, these limitations underscore the importance of controlled formulation, robust safety labeling, and cautious therapeutic use, especially in vulnerable populations, to harness camphor’s medicinal benefits while minimizing its inherent health risks.

## 10. Conclusions and Future Perspectives

This paper explores the recent advancements in understanding the therapeutic potential of *C. camphora* (camphor) within the field of phytotherapy. Throughout history, camphor has been a cornerstone in traditional medicine, revered for its ability to alleviate a variety of health issues, including pain, inflammation, and skin irritations. Its medicinal benefits extend far beyond these common uses, offering potential in the prevention and treatment of several life-threatening diseases. The rich medicinal heritage of *C. camphora* spans millennia, with its therapeutic applications rooted in multiple cultural traditions worldwide. In traditional healing systems, camphor has been used to treat a wide range of conditions, such as toothaches, urinary tract infections, and gastrointestinal discomforts like stomach irritations. These uses reflect the versatility of camphor as a remedy that addresses both acute and chronic ailments. Moreover, the medicinal properties of camphor are not limited to physical conditions; its healing capabilities extend to mental and emotional health.

Future advancement in camphor-based therapeutics is expected to center around novel, controlled-release drug-delivery systems that allow clinicians to minimize toxicity while maximizing pharmacological benefits. Techniques such as nano-encapsulation, micelle-based carriers, transdermal patches, and hydrogel matrices offer promising avenues to fine-tune absorption kinetics, sustain therapeutic levels, and avoid sudden peaks associated with toxicity. Such delivery strategies, combined with a deeper mechanistic understanding of camphor’s anti-inflammatory, antimicrobial, and neurosensory actions, could pave the way for more precise clinical indications beyond current topical and over-the-counter applications.

Simultaneously, stringent toxicological evaluations and pediatric-specific safety frameworks will be essential to mitigate the risks historically associated with accidental poisoning and misuse. Future research is also likely to explore synergistic “polyherbal” or combination therapies, where camphor is used at lower concentrations alongside complementary phytochemicals to enhance therapeutic outcomes while staying within safe exposure limits. If these scientific, regulatory, and formulation-based challenges are addressed successfully, camphor may re-emerge as a modern, evidence-driven phytomedicine, bridging traditional remedies with contemporary healthcare standards.

Despite the promising biological activities reported for camphor and camphor-containing essential oils, the majority of existing studies are based on in vitro assays, animal models, or traditional medicinal practices. These findings, while scientifically valuable, do not directly translate into clinically proven therapeutic applications. The lack of standardized dosing, pharmacokinetic data, toxicity profiling, and large-scale human clinical trials significantly limits the extrapolation of these results to clinical practice. Future research should focus on well-designed clinical studies to validate the safety, efficacy, and therapeutic relevance of camphor in human populations. Camphor represents a biologically active natural compound with diverse pharmacological properties supported primarily by experimental and traditional evidence. While current findings highlight its potential for pharmaceutical and biomedical applications, comprehensive clinical investigations are required before its therapeutic use can be conclusively established.

## Figures and Tables

**Table 1:** Bibliometric overview of camphor-related publications (2012–2021) [2,5,7,9].

Rank	Author(s)	Year	Journal	Citations	Focus Area
1	Chen et al.	2012	*Phytomedicine*	210	Antimicrobial [2]
2	Oliveira et al.	2013	*Molecules*	130	Clinical/translational studies [2]
3	Singh et al.	2014	*J. Agric Food Chem*	150	Phytochemical characterization [2]
4	Smith et al.	2015	*Molecules*	245	Pharmacology [5]
5	Gupta et al.	2016	*Int. J. Mol Sci*	172	Nanocarrier-based delivery [5]
6	Patel et al.	2017	*Phytochemistry*	145	Antimicrobial [5]
7	Kumar et al.	2018	*Journal of Ethnopharm*	198	Phytochemistry [7]
8	Lee et al.	2019	*Front. Pharmacol*	185	Anti-inflammatory mechanisms [7]
9	Wang et al.	2020	*Molecules*	160	Pharmacology [9]
10	Zhao et al.	2021	*Front. Bioeng Biotech*	138	Nanotechnology and drug delivery [9]

**Table 2:** Representative examples of leaf-derived volatile oil constituents in *Cinnamomum camphora* [11,49,50,51].

Constituent	Camphor Type	Borneol-Type	Cineol-Type	Linalool-Type
α-Pinene	27	82	7	22
Camphene	22	–	–	–
Sabinene	–	–	–	–
β-Pinene	14	22	24	–
β-Phellandrene	–	–	–	–
1p-Cineole	–	45	535	168
D-Camphor	78	–	–	50
Terpinen-4-ol	–	–	–	–
endo-Borneol	–	668	–	–
α-Terpineol	–	–	95	87
Linalool	–	–	–	266
Caryophyllene	–	–	–	33
α-Caryophyllene	–	–	–	45
γ-Elemene	–	2	–	13
Others	–	–	–	–
Total	4	393	380	992

Relative percentage composition (%) of volatile oil constituents.

## Data Availability

No new data were created or analyzed in this study.

## Abbreviations

The following abbreviations are used in this manuscript:

*Cinnamomum camphora*	Camphor
NPs	Natural products
GC-MS	Gas chromatography–mass spectrometry
FDA	Food and Drug Administration
LC_50_	Lethal concentration 50%
GPP/OPP	Geranyl diphosphate the 10-carbon monoterpene precursor
MVA	Mevalonate pathway
MEP	Methylerythritol phosphate
